# Children with cyclic vomiting syndrome: phenotypes, disease burden and mitochondrial DNA analysis

**DOI:** 10.1186/s12876-018-0836-5

**Published:** 2018-07-03

**Authors:** Ziqing Ye, Aijuan Xue, Ying Huang, Qiye Wu

**Affiliations:** Department of Gastroenterology, Children’s Hospital of Fudan University, 399 Wanyuan Road, Shanghai, 201102 China

**Keywords:** Clinical features, Cyclic vomiting syndrome, Functional gastrointestinal disorder, Genetics, Mitochondrial DNA, Pediatric

## Abstract

**Background:**

Cyclic vomiting syndrome (CVS) is characterized by repeated, stereotypical vomiting episodes. It is possibly associated with mitochondrial DNA (mtDNA) variants. We examined the phenotype, disease burden, treatment and performed mtDNA analysis in pediatric CVS.

**Methods:**

This retrospective study included 42 children with CVS in a tertiary care center. Information regarding medical history, clinical features, laboratory tests, and treatment were collected. mtDNA sequencing was performed among 13 patients.

**Results:**

Mean age of onset among patients was 4.0±3.4 years, and mean age at diagnosis was 6.7±4.2 years. CVS episodes in onset and features were stereotypic. Recognizable prodromes were reported in 54.8% patients. Neuroimaging showed previously unknown intracranial abnormalities. Gastrointestinal infection was found in four patients. Mean duration of hospitalization was 7.0±2.4 days, and mean hospitalization cost was 10,891 RMB. Sequencing showed that 4/13 patients had C16519T mtDNA polymorphism, and 2/13 patients had G3010A mtDNA polymorphism.

**Conclusions:**

Cyclic vomiting syndrome is a disabling disorder, which causes huge disease burdens to the patients and their families. Early clinical suspicion and prompt diagnosis are crucial. mtDNA polymorphisms were found in some patients, but they were not significantly associated with pediatric CVS.

## Background

Cyclic vomiting syndrome (CVS) is characterized by episodic attacks of vomiting and symptom-free interval periods [1, 2]. Prevalence of CVS is reported to be 0.3–3.8% among children based on the Rome III criteria [3–5]. About 46% patients have onset of disease before 3 years [6].

According to the Rome IV criteria [7], diagnosis of CVS requires occurrence of two or more periods of intense, stereotypical vomiting within 6 months. Because of possible underlying organic causes, appropriate medical evaluation should be performed [8].

Mitochondrial DNA (mtDNA) is maternally inherited [9], and variants adversely affect energy metabolism [10]. CVS is associated with mtDNA variants [11]. A3243G mtDNA mutation was found in a family with CVS [12]. Pediatric CVS were found to be associated with C16519T and G3010A polymorphism [10].

This study described clinical characteristics, disease burden and treatment of pediatric CVS. We also performed mtDNA sequencing among Chinese pediatric CVS patients.

## Methods

### Patient cohort

A retrospective study was conducted on patients with CVS admitted to Children’s Hospital of Fudan University from April 1st, 2008 to April 1st, 2017. Forty-two patients fulfilling the Rome IV criteria were included [1, 7]. All the patients come from unrelated families and are all of Han Chinese.

### Analysis of disease course and treatment

We examined the clinical phenotypes, disease burdens and treatment of all the patients. Data was retrieved from medical records, including demographic features, age of onset, past medical history, family history of migraine, CVS characteristics, imaging findings, laboratory results, length of stay, hospitalization costs and treatment. Imaging studies included an abdominal ultrasound, upper gastrointestinal series, and brain magnetic resonance imaging (MRI) or brain computed tomography (CT). CVS patients underwent upper and lower endoscopy. Laboratory studies included complete blood count, and chemistry panel. Metabolic tests included blood and urine tandem mass spectrometry.

### Genetic sequencing

We performed mtDNA sequencing among 13 patients. Genomic DNA was extracted from peripheral whole blood of patients. Amplification of mtDNA was performed using specific primers, and genomic DNA library was built. mtDNA sequencing resulted in an average 100× coverage using the Illumina HiSeq X ten platform. Sequence read alignments were completed using NextGene V2.3.4 against the reference genome NC_012920.1. Variants were filtered with public database after quality control, and annotated using Mitomap (www.mitomap.org). Sanger sequencing was performed with ABI3730XL and analyzed by Mutation Surveyor V4.0.8 to confirm the causal mutations with a mutant proportion higher than 10%.

### Statistical analysis

Data was analyzed using SPSS 24.0 for Windows (SPSS Inc., Chicago, IL). Continuous variables were presented as means and standard deviation, or median and inter-quartile range. Categorical variables were reported with the use of proportions and percentages.

### Ethical considerations

This study was approved by the Ethical Committee of Children’s Hospital of Fudan University. Informed consents for participation and blood sample collection were obtained from parents of the patients.

## Results

### Demographic features of patients

Of all the CVS patients, 22 were male (52.4%). Patients were born in ten different provinces in China. Five patients (11.9%) had family history of migraine, one patient had personal history of migraine, and two had family members suffering from similar cyclic vomiting attacks.

Four patients had prior history of gastrointestinal diseases, including intussusception (*n* = 2) and Henoch-Schonlein purpura (*n* = 2). One patient had nephroblastoma at 6 months of age, who received surgery and chemotherapy. One patient suffered from vasovagal syncope.

### Cyclic vomiting characteristics

The mean age of onset was 4.0±3.4 years, and mean age at diagnosis was 6.7±4.2 years. Among these patients, median duration of each attack was 3.0 (range: 2–7) days, peak episodes of vomiting per day was 10 (range: 3–30), median interval between attacks of vomiting was 30 (range: 10–90) days, and median attacks per year was 12 (range: 3–30).

Onset of CVS episodes was stereotypic among all the patients, which was characterized by onset at the same time of day. Of all the patients, 45.2% patients had symptoms in the early morning, 9.5% were awakened during midnight because of attakcs, and 45.2% patients reported episodes beginning after specific trigger events. Parents of 23 patients reported recognizable prodromes (54.8%), including abdominal pain (*n* = 11), vertigo (*n* = 5), low degree fever (*n* = 3), nausea (*n* = 3), headache (*n* = 2), weather change (*n* = 2), hiccups (*n* = 1), emotional stress (*n* = 1), and salivation (*n* = 1). All the patients had gastric contents in vomitus during attacks, bilious vomiting was present in 24 patients (57.1%), and parent-reported coffee ground vomitus in 12 patients (28.6%).

Patients suffered from other neurologic comorbidities, including paresthesia (*n* = 1) and motion sickness (*n* = 1). Twelve patients (28.6%) reported weight loss during the course. For associated gastrointestinal symptoms, 25 complained of abdominal pain, two had diarrhea, and three had constipation.

### Results of diagnostic studies

All the patients except one had neuroimaging either by brain MRI or CT. Neuroimaging showed previously unknown intracranial abnormalities in four patients, and none of them was the culprit for cyclic vomiting. Sinusitis was found in patients (*n* = 4). No abnormality was found in patients undergoing gastrointestinal series (*n* = 35). Thirty-four patients underwent abdominal ultrasonography and abdominal CT, in which two patients had mild hepatomegaly on both studies. Thirty patients received upper endoscopy. Three patients were positive for rapid urease test for *Helicobacter pylori* infection, and the other three had mild to moderate esophagitis. No abnormality was found in lower endoscopy (*n* = 14). About 34 patients undergoing electroencephalogram, and no abnormality was found. For unexpected other abnormalities, ventricular pre-excitation was present in one patient.

### Laboratory findings

Only one patient had mild anemia, and one had marginally elevated aminotransferase. One patient had marginally decreased level of ceruloplasmin. Incident gastrointestinal infection was found among patients, four were positive for serum *Helicobacter pylori* IgG, one had amoebic infection, and the other had *Blastocystis hominis* infection on stool analysis. One patient had Hepatitis B infection probably due to vertical transmission from her mother. Fifteen patients underwent blood and urine tandem mass spectrometry. Results showed slightly high urine citric acid (*n* = 1), elevated serum isovaleryl carnitine (C5) and hydroxyvaleryl carnitine (C5-OH) (*n* = 1), elevated level of urine 3-hydroxybutyric acid and acetylacetic acid (*n* = 1), low serum free carnitine (*n* = 1), and mild urine ketosis (*n* = 1).

### Treatment

During attacks, patients received fluid replacement (*n* = 25), acid suppression (*n* = 19) and ondansetron (*n* = 1) in the local hospitals according to available medical records. After being diagnosed as CVS, 26 patients has been evaluated by a pediatric psychologist in this tertiary care center. Eight patients were treated with sertraline, 10 received valproic acid, eight received sulpiride, and seven received cyproheptadine.

### Disease burden

Forty patients had multiple outpatient visits and prior hospitalizations in local hospitals because of attacks, 17 (40.5%) had been admitted for once to other hospitals, and nine (21.4%) had been admitted for more than twice to other hospitals. None of them got definite diagnosis of CVS in other hospitals. Thirty-seven patients were not local residents, and they were referred to the outpatient clinic in our center. Among all the patients, mean hospitalization cost was 10,891±3557 RMB in this center. Mean duration of hospitalization was 7.0±2.4 days.

### Analysis of mtDNA variants

Thirteen patients underwent mtDNA sequencing. Of these patients, C16519T and G3010A mtDNA polymorphism were found in 4/13 (30.8%) and 2/13 (15.4%) of the patients, respectively. An in-house database showed that the prevalence of C16519T and G3010A mtDNA polymorphism were 50.0 and 17.5%, respectively among Han Chinese subjects undergoing mtDNA sequencing. We also identified other variants, including T12338C (Case 2), A15662G (Case 3), A14693G (Case 5), C13967T (Case 7), and A4136G (Case 9). Detailed result is shown in Table 1. None mtDNA deletions had been found in the patients.

**Table 1:** Summary of mtDNA sequencing in pediatric patients with cyclic vomiting syndrome

Case	Age (years)	mtDNA sequence variants				Frequency	Associated disease	Polymorphism
1	4	*MT-CYB*	m. 15090	T > C homoplasmy	p.I115T	0.04	N/A	m.16519C > T
		*MT-CYB*	m. 15323	G > A homoplasmy	p.A193T	0.41	N/A	
2	6	*MT-RNR1*	m.1005	T > C homoplasmy	rRNA	0.38	Deafness	N
		*MT-RNR2*	m.1824	T > C homoplasmy	rRNA	0.22	N/A	
		*MT-RNR2*	m.2060	A > G homoplasmy	rRNA	0.02	N/A	
		*MT-ND4*	m.11150	G > A homoplasmy	p.A131T	0.25	N/A	
		*MT-ND5*	m.12338	T > C homoplasmy	p.MIT	0.23	Increased penetrance of deafness, LHON	
		*MT-CYB*	m.15714	C > T homoplasmy	p.S323 L	0.01	N/A	
3	6	*MT-RNR1*	m.960	C > CC heteroplasmy	rRNA	0.58	Associated with deafness	N
		*MT-ND5*	m.12361	A > G homoplasmy	p.T9A	0.59	NAFLD	
		*MT-CYB*	m.15662	A > G homoplasmy	p.I306V	0.38	Complex mitochondrial disease	
		*MT-CYB*	m. 15734	G > A homoplasmy	p.A330T	0.38	N/A	
		*MT-CYB*	m.15851	A > G homoplasmy	p.I369V	0.36	N/A	
		*MT-TT/MT-ATT*	m. 15927	G > A homoplasmy	tRNA	0.98	MS, increased penetrance of deafness, CHD	
4	13	*MT-RNR1*	m.752	C > T homoplasmy	rRNA	0.47	N/A	N
		*MT-RNR1*	m.1107	T > C homoplasmy	rRNA	0.85	N/A	
		*MT-ND4*	m. 12026	A > G homoplasmy	p.I423V	0.51	Diabetes mellitus	
5	13	*MT-TE*	m.14693	A > G homoplasmy	tRNA	0.65	MELAS, LHON, deafness, HTN	N
		*MT-CYB*	m. 14766	C > G homoplasmy	p.T7I	0	N/A	
6	6	*MT-RNR1*	m.1382	A > C homoplasmy	rRNA	0.42	N/A	m.3010 G > A
		*MT-TC*	m.5802	T > C homoplasmy	rRNA/tRNA	0	increased penetrance of deafness,	
		*MT-C01*	m.6259	A > G heteroplasmy	p.E119G	0	N/A	
7	10	*MT-ATP6*	m.8842	A > G homoplasmy	p.I106V	0.13	N/A	N
		*MT-C03*	m.9319	A > G homoplasmy	p.H38R	0	N/A	
		*MT-ND3*	m. 10327	C > T homoplasmy	p.S90 L	0.02	N/A	
		*MT-ND5*	m. 13967	C > T homoplasmy	p.T544 M	0.32	Associated with LHON	
8	14	*MT-RNR2*	m.1715	C > T homoplasmy	rRNA	0.40	N/A	N
		*MT-ND2*	m.5277	T > C homoplasmy	p.F270 L	0.26	N/A	
		*MT-C02*	m.7980	A > G homoplasmy	p.D132G	0.01	N/A	
		*MT-ATP6*	m.8945	T > C homoplasmy	p.M140 T	0.03	N/A	
		*MT-TP/MTATT*	m. 15968	T > C homoplasmy	tRNA	0.42	N/A	
9	7	*MT-RNR1*	m.1147	G > A heteroplasmy	rRNA	0	N/A	m.16519C > T
		*MT-ND1*	m.4136	A > G heteroplasmy	p.Y277C	0.12	LHON	
		*MT-ND2*	m.4638	A > G homoplasmy	p.I57V	0.01	N/A	
		*MT-ND2*	m.4833	A > G homoplasmy	p.T122A	0.82	Associated with diabetes, Alzheimer, Parkinson disease	
10	11	*MT-RNR2*	m.2417	C > G homoplasmy	rRNA	0.02	N/A	m.16519C > T
		*MT-TL1*	m.3290	T > C homoplasmy	tRNA	0.2	Possibly associated with HTN	
		*MT-TQ*	m.4345	C > T homoplasmy	tRNA	0.01	Possibly associated with HTN	
		*MT-ND2*	m.5263	C > T homoplasmy	p.A265V	0.57	N/A	
		*MT-TH*	m.12153	C > T homoplasmy	tRNA	0.09	N/A	
11	12	*MT-RNR1*	m.1520	T > C heteroplasmy	rRNA	0.06	N/A	m.16519C > T, m.3010 G > A
		*MT-RNR2/MT-RNR3*	m.3206	C > T heteroplasmy	rRNA	0.40	N/A	
		*MT-ND2*	m.5466	A > G homoplasmy	p.T333A	0.06	N/A	
		*MT-TG*	m.9992	C > T homoplasmy	tRNA	0.02	N/A	
		*MT-ND5*	m.13834	A > G homoplasmy	p.T500A	0.09	N/A	
		*MT-CYB*	m. 14979	T > C homoplasmy	p.I78T	0.44	N/A	
12	7	*MT-RNR1*	m.961	T > C heteroplasmy	rRNA	0.99	Deafness, possibly associated with NVM	N
		*MT-RNR2*	m.1709	G > A homoplasmy	rRNA	0.36	N/A	
13	3	*MT-HV2/ MT-OHR/ MT-TFY/ MT-CSB2/ MT-ATT/ MT-CR*	m.301	A > ACC heteroplasmy	Non-coding	0.00	N/A	N
		*MT-ND1*	m.4029	C > T homoplasmy	p.12411	0.01	N/A	
		*MT-ND1*	m.4086	C > T homoplasmy	p.V260 V	0.75	N/A	
		*MT-C02*	m.8149	A > G homoplasmy	p.R188R	0.32	N/A	
		*MT-C03*	m.9548	G > A homoplasmy	p.G114G	0.97	N/A	
		*MT-C03*	m.9944	T > C heteroplasmy	p.D246D	0.17	N/A	
		*MT-HV1/ MT-TAS2/ MT-ATT/ MT-CR/ MT-7SDNA*	m.16108	C > T homoplasmy	Non-coding	0.24	N/A	

Frequency: reported in Gene Bank, *HTN* hypertension, *LHON* Leber hereditary optic neuropathy, *MELAS* mitochondrial encephalomyopathy with lactic acidosis and stroke-like episodes, *MS* multiple sclerosis, *N/A* not available, *NVM* noncompaction of ventricular myocardium, *N* none identified, Polymorphism: reported to be associated with CVS

## Discussion

There is a paucity of data about pediatric CVS in China. To our knowledge, this is the largest clinical study and genetic analysis on mtDNA of pediatric CVS in China.

In this study, patients were diagnosed as pediatric CVS fulfilling the Rome IV criteria. They had two or more periods of intense, unremitting nausea and paroxysmal vomiting within a six-month periods. These episodes are stereotypical and interspersed with symptom-free interval periods [7]. According to the 2008 Consensus Statement on the Diagnosis and Management of Cyclic Vomiting Syndrome by North American Society for Pediatric Gastroenterology, Hepatology, and Nutrition, pediatric CVS generally requires at least five episodes of vomiting in a year [8]. There were statements which criticized the minimum of two attacks in both the Rome III and Rome IV criteria for lacking specificity [2]. However, studies utilizing the Rome III and IV criterion did not show significantly higher prevalence rates [4, 13]. Based on these data and the impact of CVS attacks for the quality of life of children and their families, the Rome IV working group decided that early diagnosis is important and left the minimum number of two episodes in CVS diagnosis unchanged [2, 7]. Over the last decade, there have been advancements in the pathogenesis and diagnostic criteria in pediatric CVS [14]. Therefore, we utilized the latest Rome IV in this study.

Our study showed a mean onset of 4.0±3.4 years among patients. They had recognizable prodromes and stereotypical attacks. These results were consistent with other studies on CVS in Chinese children [15–18]. Reports involving patients in other populations also showed a similar pattern of disease onset and vomiting characteristics [19, 20]. However, our study showed that only 11.9% of patients had family history of migraine and 2.3% had personal history of migraine. The frequency of personal and/or family history of migraine in this study seemed lower than that reported in western populations [21], which was about 39–82% [22, 23]. Dong et al. reported that personal migraine history was seen only in 7.3% of Chinese children with CVS [18]. Prevalence of migraine in the general Chinese population was 9.3% in a nationwide survey [24], compared to 14.9% in United States [25]. Study found that under-diagnosis and misdiagnosis of migraine were common in China, which might be an explanation for the low frequency of personal/family history of migraine among our cohort [26].

Moreover, parent-reported coffee-ground vomitus was present in 12 (28.6%) of subjects during attacks. All of them were treated in emergency departments in local hospitals with hydration and supportive care, and half received gastric acid suppressants. Therefore, there was no delay in medical care during attacks among them. However, abortive therapy was only instituted in one patient with ondansetron. After admission to our institution, ten underwent upper endoscopy, and only one was diagnosed with esophagitis. Repetitive nausea, vomiting, and retching in severe cases might cause hematemesis because of mucosal tear (Mallory-Wise syndrome) [27]. Pediatric CVS might be under-diagnosed and misdiagnosed in local hospitals, simply as “gastritis, food poisoning” due to physicians’ inadequate knowledge about pediatric functional gastrointestinal disorders. Therefore, delay in diagnosis was common [22, 28]. Lack of abortive care might lead to prolonged emetic phases and subsequent mucosal tear in our patients, which maybe the explanation for high frequency of parent-reported coffee ground vomitus. Upper endoscopy after admission ruled out severe mucosal diseases.

There is no randomized controlled trial on treatment of CVS. Retrospective studies showed favorable clinical responses with cyproheptadine [29], propanolol and pizotifen [30]. Valproate is also considered as an effective prophylactic therapy for pediatric CVS [31]. Patients admitted to this tertiary care center generally received prophylactic medications, including cyproheptadine, valproate acid, or both. More than half of the patients had been consulted by a pediatric psychologist. Integration of psychotherapy is beneficial, as psychologic factors are associated with functional gastrointestinal disorders including CVS [32]. However, most patients referred from local hospitals in this study did not receive prophylactic therapy and multidisciplinary treatment involving pediatric psychologists before admission.

The C16519T polymorphism locates in the non-coding mtDNA control region, and is six-fold more commonly seen in pediatric CVS than in controls [10]. The G3010A polymorphism locates in the 16S-ribosomal RNA gene [10], which increases the odds ratio for CVS by 17-fold in subjects with C16519T. These two polymorphisms are not found in adult CVS patients [33]. Han et al. performed mtDNA sequencing among four Chinese children with CVS plus, (CVS with + neurocognitive disorders), in which two had A3243G mutation and two had C16519T polymorphism [15]. In this study, none A3243G mutation has been identified, 4/13 (30.8%) patients had C16519T, and 2/13 (15.4%) had G3010A polymorphism. However, considering the relatively high prevalence of C16519T and G3010A polymorphism from an in-house database of Chinese subjects and lack of frequency of these polymorphisms among large cohort of healthy controls, it is not sufficient to detect significant associations among mtDNA polymorphism with Chinese pediatric CVS patients.

In the study, there were mtDNA variants among patients associated with other diseases. Homoplasmic A15662G mutation was identified in case 3, which was previously reported in a patient with complex mitochondriopathy [34]. However, none functional analysis had been performed, and three other variants were identified in this patient (T3398C, T4216C, and G15812A) [34]. This subject did not have manifestations of mitochondrial diseases. Heteroplasmic A4136G mutation was identified in case 9, which was previously reported in a family with Leber hereditary optic neuropathy (LHON) [35]. Other mtDNA mutations were also found in these patients [35]. In another study, A4136G was described in pediatric patients with mitochondrial encephalopathies [36]. Patients harboring variants A4136G did not have LHON, but present with clinical symptoms of mitochondrial disease [36].

Because of repeated visits to emergency department during attacks and diagnostic studies to rule out organic causes, there were substantial disease burdens caused by CVS to patients and families. A national study among adults in United States showed that there were 20,952 CVS hospitalizations during a two-year period, and caused about 400 millions US dollars of hospital charges [23]. Emergency department visits were 2.3±2.0 per year among pediatric CVS patients [37]. It also imposes a major impact on quality of life [38]. Patients generally have lower health-related quality of live than healthy controls [39]. Failure to recognize CVS occurred in 93% of emergency department visits in previous report [40], which was consistent in this study. None of our patients were diagnosed as CVS in other hospitals. Also, 40 patients had multiple outpatient visits and prior hospitalizations, which further increased the disease burden.

However, our results should be interpreted with caution. This was a retrospective study from a single tertiary care center. Only 13 patients underwent mtDNA sequencing due to the retrospective setting, and mtDNA from mothers of the patients were not available. This study cannot indicate associations between mtDNA polymorphisms with pediatric CVS. Moreover, there was possible selection bias, as only cases with severe cyclic vomiting attacks had been admitted and included in the study. A prospective study involving larger number of subjects and a cohort of healthy controls from the same populations are needed to confirm the relationship between mtDNA variants and CVS.

## Conclusions

Pediatric CVS is a chronic and debilitating disease causing considerable disease burdens to patients and family. Early clinical suspicion and prompt diagnosis by pediatricians enable patients to receive adequate symptom-tailored management.

## Abbreviations

CT: Computed tomography
CVS: Cyclic vomiting syndrome
LHON: Leber hereditary optic neuropathy
MRI: Magnetic resonance imaging
mtDNA: Mitochondrial DNA
